# A surgical approach to giant condyloma (Buschke-Löwenstein tumour) with underlying superficial vulvar carcinoma: A case report

**DOI:** 10.3892/ol.2012.1027

**Published:** 2012-11-14

**Authors:** JOSKO ZEKAN, DAVOR PETROVIC, SAMER EL-SAFADI, MAJA BANOVIC, DAVOR HULINA, ZLATKO HRGOVIC

**Affiliations:** 1 Departments of Gynaecological Oncology, University Medical School, 10000 Zagreb, Croatia;; 2 Gynaecological Pathology, University Medical School, 10000 Zagreb, Croatia;; 3 Department of Gynaecology and Obstetrics, University Hospital Giessen and Marburg GmbH, Giessen D-35392, Germany;; 4 Department of Plastic and Reconstructive Surgery, University Medical School, 10000 Zagreb, Croatia;; 5 Hospital of Gynaecology and Obstetrics, Offenbach GmbH, Offenbach D-63069, Germany

**Keywords:** giant condyloma, Buschke-Löwenstein tumour, vulvar carcinoma, surgery

## Abstract

Anogenital warts (condyloma acuminatum or venereal warts) are a common sexually transmitted disease in males and females. Common clinical treatment of anogenital warts is conservative, however, in extreme cases conservative therapy is insufficient and surgical excision is required. Giant condyloma acuminata (Buschke-Löwenstein tumour) is an extremely rare clinical type of genital wart, characterised by aggressive down growth into underlying dermal structures. A 55-year-old female presented with cauliflower-like growth over the anogenital and sacral region, earlier diagnosed as condyloma acuminatum which was resistant to conservative therapy. During the period between 2005 and 2008 the patient underwent five surgical procedures. Due to the size and location of the tumour, gynaecological and plastic surgeons were involved in the procedures. In addition, definitive histology examination identified a superficial vulvar carcinoma.

## Introduction

Anogenital warts (condyloma acuminatum or venereal warts) are a common sexually transmitted disease among females and males (1,2). The causal role of human papillomaviruses (HPV) in anogenital wart formation has been firmly established biologically and epidemiologically (3,4). Genital HPV infections are transmitted primarily through sexual contact, with a lifetime risk of 50–80% (5). The highest rate of genital HPV infection has been identifed in adults between 18 and 28 years of age (6,7). The immune system effectively repels the majority of HPV infections and is associated with marked localised cell mediated immune responses. However, approximately 10% of individuals develop a persistent infection, with risk of developing benign proliferative lesions, high-grade precursors and eventually invasive carcinomas (8). HPVs are classified into high- or low-risk types depending on oncogenic potential. Low-risk types 6 and 11 are isolated in approximately 90% of genital wart cases (3). The most common clinical treatment is conservative, with local chemical or physical destruction and immunological therapy (9). In more extreme cases conservative therapy is insufficient and surgical excision is required.

Giant condyloma acuminata (GCA; Buschke-Löwenstein tumour) is an extremely rare clinical form of genital warts, characterised by aggressive down growth into underlying dermal structures (10,11). A complex histological pattern may exist with areas of benign condyloma intermixed with foci of atypical epithelial cells or well differentiated squamous cell carcinoma. GCA is mainly localised to the genital region, however, in rare cases the tumour is localised to distinct histological zones of the anorectal region. Due to infiltration of the underlying tissue, fistulae and abscesses may be observed. GCA is resistant to chemotherapy or radiotherapy and usually requires local radical resection for curative treatment. The study was approved by the ethics committee of University Medical School, Zagreb. Written informed consent was obtained from the patient.

## Case report

**Figure 1.**
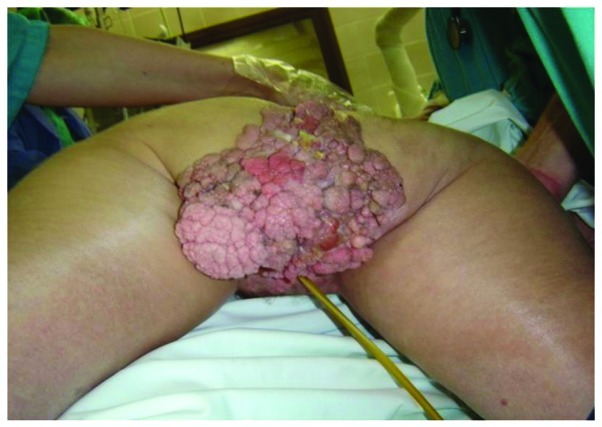
Patient with giant condyloma prior to reconstructive surgery.

A 55-year-old female presented with cauliflower-like growth over the anogenital and sacral region. The growth had been diagnosed previously as condyloma acuminatum which was resistant to conservative therapy (Fig. 1). The patient’s medical history was as follows: at 17 years old the patient was diagnosed with infective mononucleosis and 6 months later with viral pneumonia. In 1979, the patient suffered from pyelonephritis caused by *E. coli*, with subsequent unilateral permanent kidney lesion. Multiple condyloma were diagnosed for the first time during the patient’s first pregnancy in 1970, at the perineal surface and were surgically removed in the same year, following delivery. Four years following excision recurrence was identified and was treated successfully with albothyl solution for two months.

During the first trimester of the patient’s second pregnancy (1978) warts appeared for the third time with altered clinical presentation; spread across the entire anogenital region (perineum, anal orifice and labia majora) and became multilayered and painful. Despite repeated albothyl therapy, growth continued. Prior to labour the warts were removed by electro-cauterisation and the whole surface was treated locally with interferon (IFN) ointment. During the following year there were no visible warts. Between 1980 and 1981, due to recurrence, the patient underwent two additional surgical procedures followed by IFN treatment. Positive response to treatment lasted for 6–8 months and was followed by five excision procedures under local anaesthesia between 1983 and 1984. All condyloma were removed. The severity of the disease increased the following year and was successfully treated with IFN ointment over one year. In 1986, tumour size increased again. The patient recieved local IFN therapy, however, treatment response was inadequate as the tumour was reduced in size by 50%. Condyloma size remained constant until 2003 when the patient entered menopause. Podophyllin treatment was administered, however, the side-effects included bilateral inguinal lymphadenopathy and marked pain. At this point the patient was admitted to our unit.

**Figure 2:.**
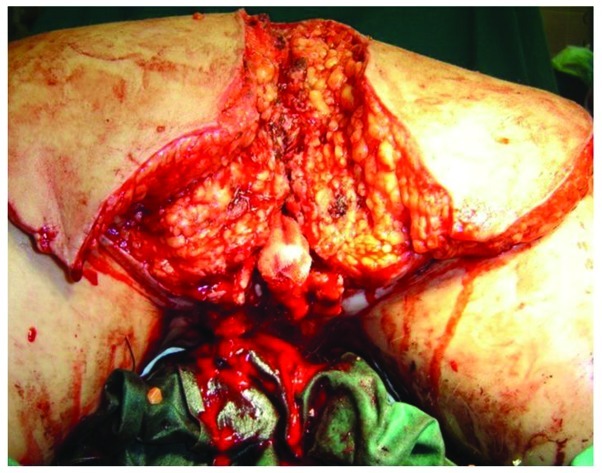
Patient during the surgical proceedure.

**Figure 3:.**
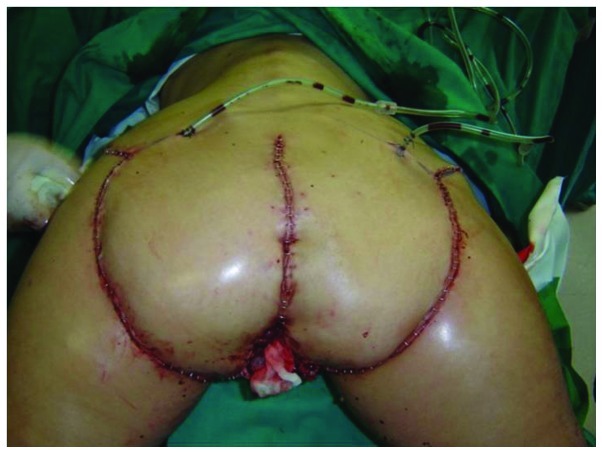
Patient directly following surgery.

Between 2005 and 2008 the patient underwent five surgical procedures. The procedures were performed by gynaecological and plastic surgeons due to the size and location of the tumour. The first surgery was a loop colostomy on the sigmoid colon performed by an abdominal surgeon. The following procedures were performed by a plastic and reconstructive surgeon over four surgical periods; radical excision of the vulvar, perineal, anal and sacral condyloma with the preservation of urethral orifice, vagina and anus (Fig. 2). Reconstruction of the dorsal defect was formed using two large fascio-cutaneal flaps based on the superior gluteal artery. The remaining defect of the vulva was reconstructed by local transpositional fascio-cutaneal flaps from the medial side of the upper thighs. Following the third surgery, a postoperative infection developed with partial dehiscence of the local transpositional flaps from the upper thighs, therefore, necrectomy was performed, covering the residual with a split thickness skin graft from the right upper thigh. Seven months following, excision of the cicatrices was performed due to development of contractures of the perineal skin and the defects split thickness skin graft was utilised for covering. The final surgery was the occlusion of the colostomy performed by the abdominal surgeon (Fig. 3).

**Figure 4.**
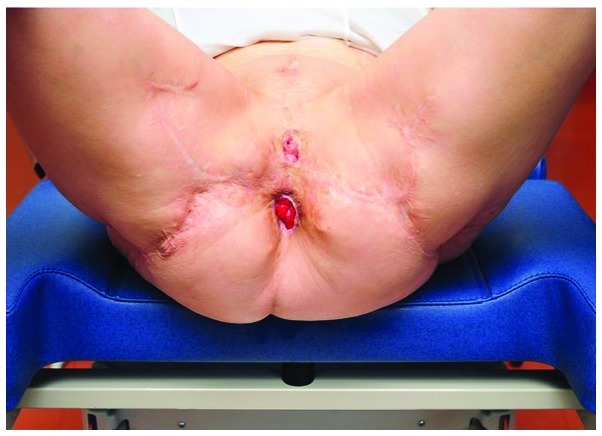
Patient following the healing process.

Following 6 months, local status was a preserved urethral orifice and vaginal introitus with a small rectal mucosa prolapse (Fig. 4).

**Figure 5.**
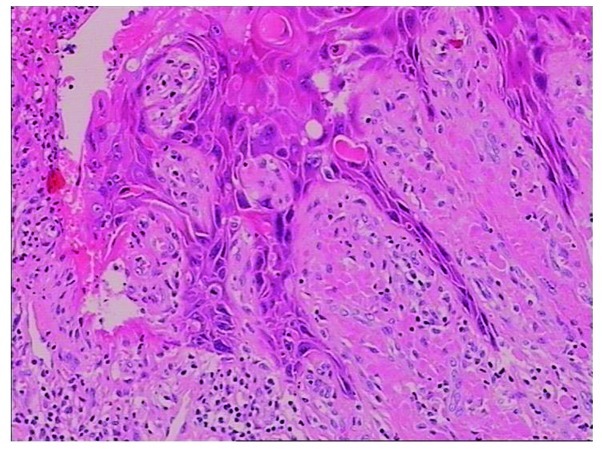
Histology of the giant condyloma.

In the present case, the giant condyloma appeared to exhibit characteristics of a normal condyloma acuminatum and a superficial planocellular carcinoma. The majority of the material received for pathology analysis was condyloma accuminatum with prominent acanthosis, dyskeratosis, hyper-keratosis and prominent granular layer. In superficial epithelial cells typical perinuclear cytoplasmic ‘halos’ and pyknotic or slightly enlarged nuclei were observed and bi-nuclear cells were present. In specific areas invasive superficial squamous carcinoma was identified with invasion of the underlaying dermis with small clusters of cells accompanied with prominent mononuclear inflammatory infiltrate. Immunohistochemical analysis with p16 monoclonal antibody (clone E6H4 against p16 protein; CINtec Histology; Roche Diagnostics GmbH, Mannheim, Germany) revealed positivity in tumour cells. Additional analysis using Digene Hybrid Capture 2 (Qiagen, Hilden, Germany) was performed and the presence of HPV genotype 6 and 11 was confirmed (Fig. 5).

## Discussion

Anogenital warts are the most common outcome of HPV genital infection. Therapeutics against this sexually transmitted disease are currently associated with low efficacy, due to a 30–70% recurrence rate identified six months following therapy administration (9). In rare cases, anogenital warts develop into extremely large tumour masses leading to deterioration of patient quality of life. An identified underlying histopathology of specific cases of giant condyloma is superficial planocellular carcinoma. Patients with high susceptibility to local development and fast progression (in growth and malignancy) and the highest rate of recurrence often exhibit various types of immunodeficiency. In addition, immunodeficiency leads to difficulties in evaluation of optimal therapeutic management. However, patients with no marked immunodeficiency and treatment-resistent genital warts have been identified. Furthermore, condyloma lesions occasionally form large exophytic masses, interfering with intercourse, normal urination, defecation or vaginal delivery.

Commonly, GCA develops as cauliflower-like masses and the tumours exhibit histological features of pseudo-epitheliomatous profileration and local invasion. In the absence of metastases, they are termed Buschke-Löwenstein tumours. Due to the aggressive local development of these masses they belong to the verrucous carcinoma group, although a malignant histological alteration in the form of micro-invasive carcinoma or well-differentiated epidermoid keratinising carcinoma has been reported. Due to the high frequency of local recurrence, radical surgical excision is the current treatment of choice as topical preparations and chemotherapy are generally considered ineffective (12). The method selected for reconstruction is crucial, particularly in neglected cases similar to the present case study. Local tissue availability and the patient’s condition and attitude towards the development of the disease are major factors for reconstruction with local fascio-cutaneous flaps. Five years following surgury the present patient is disease-free with no recurrence.
